# Effect of core versus enhanced implementation strategies on adherence to a clinical pathway for managing anxiety and depression in cancer patients in routine care: a cluster randomised controlled trial

**DOI:** 10.1186/s13012-023-01269-0

**Published:** 2023-05-22

**Authors:** Phyllis Butow, Mona M. Faris, Joanne Shaw, Patrick Kelly, Sharon He, Marnie Harris, Jessica Cuddy, Lindy Masya, Liesbeth Geerligs, Brian Kelly, Afaf Girgis, Nicole Rankin, Philip Beale, Thomas F. Hack, Laura Kirsten, Haryana Dhillon, Peter Grimison, Rosalie Viney, Josephine M. Clayton, Timothy Schlub, The ADAPT Program Group, Toni Lindsay, Melanie Lovell, Tim Luckett, Michael Murphy, Jill Newby, Don Piro, Melanie Price, Tim Shaw, Jackie Yim, Heather L. Shepherd

**Affiliations:** 1 School of Psychology, Psycho-Oncology Co-operative Research Group (PoCoG), The University of Sydney, 31 Ellalong Rd North Turramurra 2074, Sydney, NSW Australia; 2 School of Public Health, Faculty of Medicine and Health, The University of Sydney, Sydney, NSW Australia; 3 School of Medicine and Public Health, University of Newcastle, Newcastle, NSW Australia; 4 South West Sydney Clinical Campuses, UNSW Medicine & Health, University of New South Wales, Kensington, Australia; 5 Faculty of Medicine and Health, The University of Sydney, Sydney, NSW Australia; 6 Department of Medical Oncology, Concord Hospital, NSW, Concord, Australia; 7 College of Nursing, Rady Faculty of Health Sciences, University of Manitoba, Winnipeg, Manitoba Canada; 8 Nepean Hospital, Nepean Cancer Care Centre, Kingswood, NSW Australia; 9 Chris O’Brien LifehouseCamperdown, Sydney, NSW Australia; 10 Centre for Health Economics Research and Evaluation, University of Technology, Sydney, NSW Australia; 11 HammondCare, The Palliative Centre, Greenwich Hospital, Greenwich, NSW Australia; 12 Northern Clinical School, Faculty of Medicine and Health, The University of Sydney, Sydney, NSW Australia; 13 Susan Wakil School of Nursing and Midwifery, Faculty of Medicine and Health, The University of Sydney, Sydney, NSW Australia

**Keywords:** Implementation strategies, Cluster randomised controlled trial, Anxiety and depression management

## Abstract

**Background:**

Optimal strategies to facilitate implementation of evidence-based clinical pathways are unclear. We evaluated two implementation strategies (Core versus Enhanced) to facilitate implementation of a clinical pathway for the management of anxiety and depression in cancer patients (the ADAPT CP).

**Methods:**

Twelve cancer services in NSW Australia were cluster randomised, stratified by service size, to the Core versus Enhanced implementation strategy. Each strategy was in place for 12 months, facilitating uptake of the ADAPT CP (the intervention being implemented). The Core strategy included a lead team with champions, staff training and awareness campaigns prior to implementation, plus access to feedback reports and telephone or online support during implementation. The Enhanced strategy included all Core supports plus monthly lead team meetings, and proactive, ongoing advice on managing barriers, staff training and awareness campaigns throughout implementation*.* All patients at participating sites were offered the ADAPT CP as part of routine care, and if agreeable, completed screening measures. They were allocated a severity step for anxiety/depression from one (minimal) to five (severe) and recommended management appropriate to their severity step. Multi-level mixed-effect regression analyses examined the effect of Core versus Enhanced implementation strategy on adherence to the ADAPT CP (binary primary outcome: adherent ≥ 70% of key ADAPT CP components achieved versus non-adherent < 70%), with continuous adherence as a secondary outcome. Interaction between study arm and anxiety/depression severity step was also explored.

**Results:**

Of 1280 registered patients, 696 (54%) completed at least one screening. As patients were encouraged to re-screen, there were in total 1323 screening events (883 in Core and 440 in Enhanced services). The main effect of implementation strategy on adherence was non-significant in both binary and continuous analyses. Anxiety/depression step was significant, with adherence being higher for step 1 than for other steps (*p* = 0.001, OR = 0.05, 95% CI 0.02–0.10). The interaction between study arm and anxiety/depression step was significant (*p* = 0.02) in the continuous adherence analysis only: adherence was significantly higher (by 7.6% points (95% CI 0.08–15.1%) for step 3 in the Enhanced arm (*p* = .048) and trending to significance for step 4.

**Discussion:**

These results support ongoing implementation effort for the first year of implementation to ensure successful uptake of new clinical pathways in over-burdened clinical services.

**Trial registration:**

ANZCTR Registration: ACTRN12617000411347 (Trial registered 22/03/2017; https://www.anzctr.org.au/Trial/Registration/TrialReview.aspx?id=372486&isReview=true)

**Supplementary Information:**

The online version contains supplementary material available at 10.1186/s13012-023-01269-0.

Contributions to the literature

- Various implementation strategies have been shown to promote implementation efforts, but the optimal combination and dose of implementation strategies is not well researched
- This study sought to determine the optimal dose of implementation effort (Core versus Enhanced) required to achieve adherence to an anxiety/depression clinical pathway (ADAPT CP).
- Enhanced implementation effort assisted services in responding to patients with moderate levels of anxiety and/or depression.
- Data suggests that sufficient staff and resources are required to enable successful implementation.
- These results provide some of the first empirical data on the extent and duration of implementation effort required to support health service change.

## Introduction

Clinical pathways (CPs) are standardised, evidence-based multidisciplinary management plans, which identify an appropriate sequence of clinical interventions, timeframes, milestones and expected outcomes for one or more patient groups [1]. By their operational nature, CPs provide a level of detail over and above that provided in clinical guidelines. CPs are increasingly used in healthcare to achieve optimal, evidence-based and cost-effective outcomes, [2, 3] increase hospital efficiency [4, 5], decrease operational costs [6, 7], reduce lengths of stay [8], and decrease mortality rates [9].

However, while CPs have been shown to improve patient outcomes, this is not always the case [10], possibly due to poor uptake or incomplete adherence to CPs resulting from environmental, system or practitioner barriers [11, 12]. Deviations from CPs may reflect beneficial tailoring to the needs of individual patients but may also result in reduced quality of patient care, [5, 13] increased staff burden due to additional steps required to address deficiencies [3], and increased staff resistance to future health service change.

Due to these negative outcomes, increasing research effort has explored strategies to promote uptake and adherent use of health service interventions [14], culminating in sophisticated, evidence-based implementation frameworks, such as the Promoting Action Research in Health Services framework (PARiHS) [15] and the Consolidated Framework for Implementing Research (CFIR) [16]. These frameworks propose that, along with evidence, context and intervention characteristics, facilitation is key to implementation success. Facilitation encompasses resources and processes offered both internally and by the research team, to support staff in implementing interventions such as CPs. While there is increasing understanding of the nature of facilitation strategies, there is very little clarity regarding the optimal dose of facilitation required. Without such data it is difficult for health systems to plan implementation efforts when introducing new interventions into health systems, including CPs, into routine care.

Our group developed a CP for screening, assessment and management of anxiety and depression in adult cancer patients (the ADAPT CP) to guide best practice in Australia [17]. The ADAPT CP is based on evidence review, refined through stakeholder interviews [18] and a Delphi consensus process [19]. The ADAPT CP follows a stepped care model incorporating iterative screening at recommended intervals, with triage to one of five steps, each with a recommended management plan (from universal care and self-management for those with minimal or mild levels of anxiety and/or depression, to specialist care for those with severe anxiety and/or depression), with review and change in step where necessary. Evidence-based recommendations on staff responsibilities, content and timing of interventions, are provided for each step and tailored to available resources [17]. Drawing on the PARiHS and CFIR frameworks to support CP implementation, we developed an online portal (the ADAPT Portal) [20] to operationalise as many processes as possible, increase efficiency and reduce staff time and burden. We also developed staff education modules [21], patient information, and an online cognitive-behavioural intervention for mild to moderate anxiety and depression [22] to address concerns raised by health professionals in an earlier barrier analysis [18].

Our team has reported extensively on implementation preparation, processes and outcomes of the ADAPT trial [23], including organisational readiness for change [24], identification of barriers and facilitators [25], selection and definitions of evaluation outcomes [26], staff perspectives on acceptability and appropriateness [27] and tailoring of implementation processes according to site needs [28]. Forthcoming publications will address implementation costs and fidelity, sustainability of the ADAPT CP and effect of the ADAPT CP on health service use.

In this cluster trial, our primary objective was to determine whether “dose” of implementation strategy (Core versus Enhanced) effects staff adherence to the ADAPT CP. Our secondary objective was to examine the effect of step allocation (overall, and by implementation support arm) on total and component adherence scores.

Our primary hypothesis was that the proportion of eligible patients screened, for whom sites achieve acceptable adherence to the ADAPT CP (defined as yes: having completed ≥ 70% of the recommended ADAPT CP components versus no.: < 70%) would be greater in the Enhanced implementation strategy arm than in the Core implementation strategy arm.

Secondary hypotheses included that:

1. Adherence scores for each component of the ADAPT CP would be greater in the Enhanced implementation strategy arm than in the Core implementation strategy arm.
2. As more actions are required of staff for higher (more severe) steps of anxiety/depression, adherence would be lower for higher than for lower steps of anxiety and depression.
3. There would be a significant interaction between step and implementation strategy arm, with better adherence to higher steps of anxiety and depression in the Enhanced implementation strategy arm than that in the Core implementation support arm.

## Methods

### Study design and setting

This study was a mixed-methods, cluster randomised controlled trial (CRCT) which was conducted from January 2017 to December 2020. Cancer services in New South Wales (NSW), Australia’s most populous state, were invited to participate in the study. Participating services were randomised to a Core versus Enhanced implementation strategy to promote uptake of and adherence to the ADAPT CP. Randomisation was stratified by size of service (large (≥ 100 new pts./year) versus small (< 100 new pts./year)) to ensure approximately equal patient volume in the two study arms.

### Service inclusion criteria

Eligible cancer services could operate within the public and/or private healthcare systems, and be whole cancer services or single, independent departments within services, such as tumour streams (e.g., breast cancer or haematology) or treatment streams (such as medical or radiation oncology). Care was taken to ensure representation of services located in both major cities and regional areas.

### Recruitment: cancer services, staff and patients

Researchers met with cancer service directors and multidisciplinary representatives to discuss the study in detail. Following confirmation of participation, a local ADAPT champion, who would lead implementation of the ADAPT CP at the cancer service was appointed to liaise with the research team, and facilitate engagement with the service workforce. Services also nominated a lead team to engage with the research team, optimally comprised of multi-disciplinary representatives including medical, nursing, psychosocial, administrative and IT staff, the identified ADAPT CP champion(s), and other opinion leaders as required. Cancer service staff involved in any way with implementation of the ADAPT CP were provided with participation information sheets by researchers, with consent recorded either by completion of an online survey or a consent form.

All patients at participating study sites were offered care in accordance with the ADAPT CP as part of routine care during the 12-month implementation period. Patients who agreed to complete screening within the ADAPT Portal were invited to participate in the study and give informed consent for researchers to access their medical records and health care utilisation.

### Study procedure

Study processes have been described in detail elsewhere [23]. The ADAPT Cluster RCT involved different stages, which included an Engagement period, with scheduled time (approximately 2 months) to prepare cancer services for implementation of the ADAPT CP; Go-Live, when the ADAPT Portal was launched; and the Implementation period, which comprised 12 months of supported implementation where the ADAPT CP was used by cancer services as part of routine care. The Engagement period (identical for all participating services) comprised 6–8 engagement meetings with the lead team, facilitated by the ADAPT research team, to tailor the ADAPT CP and Portal to local preferences, workflow and resources. Cancer service staff were invited to attend face-to-face information and education sessions about the study processes, the ADAPT CP and the ADAPT Portal, and were provided with access to online education modules on how to introduce and conduct screening for anxiety/depression, triage screening results, and make referrals if needed.

### Randomisation and data collection measures

Randomisation occurred at the end of the Engagement Period, with sites randomised in blocks of 4 to the Core or Enhanced intervention group, stratified by size (large vs. small). Allocation concealment was preserved for study sites and staff throughout the study. Service staff completed baseline (T0) questionnaires prior to randomisation and at T1 (6 months into implementation) and T2 (study close, after 12 months of implementation). A subsample also participated in semi-structured interviews at these timepoints. The purpose of questionnaires and interviews was to gather information about staff and organisational readiness, staff perception of the ADAPT CP such as its usefulness, appropriateness, and potential and actual barriers to implementation (these data are reported elsewhere [24–28]). ADAPT staff conducting staff interviews and statistical analyses were blinded to site allocations.

### Intervention arms

Implementation strategies are outlined in Table 1. *Core* strategies (delivered to both study arms) were consistent with usual roll out of a CP in the Australian NSW health context. Lead team members guided tailoring of the ADAPT CP and Portal to fit the local context, to increase ownership and maximise intervention fit; awareness campaigns were run as the ADAPT CP was launched, including posters and presentations; staff received relevant training and access to education modules to increase self-efficacy and readiness; and champions were provided monthly ADAPT Portal activity audit and feedback reports and additional support from the ADAPT team at their request (a passive approach).

**Table 1:** Implementation strategies as delivered according to randomisation^a^

	Both Core and Enhanced implementation strategies arms	Enhanced implementation strategies arm *only*
**Strategy**		
Awareness campaign	•Roadshow presentation by ADAPT staff at the site 8 weeks before “go-live” •Poster displayed prominently 4 weeks prior to and at “go-live,” (T0) •Email from site champion to all staff 1 week before “go-live” (T0)	•Additional posters at 4-monthly intervals during implementation •Newsletters emailed to site staff at 2, 4, 6, 9, and 12 months
Champions	•Champion identification and role definition •Provision of email templates for champion to send staff •Inclusion of champion contact details in all staff communication	•Additional proactive contact with Champions at monthly intervals to discuss progress, provide audit reports and discuss additional implementation strategies as needed
Staff and patient Education	•Health professionals training: •Portal training + user guides •ADAPT Clinical pathway •Patient information: •Anxiety and depression	•Proactive training if required for new staff over year of implementation
Academic detailing and support	•Tailoring of the ADAPT portal to site requirements during engagement meetings •Champions provided with a written report summarising change readiness data from staff interviews at T0, T1, T2 •ADAPT telephone and email support line, available thoughout study period •Study close meeting with all key staff to discuss sustainability of the ADAPT CP	•Monthly face-to-face meetings using standing items (1) monthly ADAPT Portal activity data presented alongside written report, (2) Local lead team updates, (3) Portal functionality (4) Staff changes/training needs training needs (5) Local issues for highlighting in newsletter (6) Sustainability (7) Other locally rained issues •Opportunity to discuss written report summarising change readiness data from staff interviews at T0, T1, T2 •Quarterly review of ADAPT portal configuration to confirm allocated responsibilities and service tailoring
Reporting	•Monthly written reports on ADAPT Portal activity data	•Report presented face-to-face by research team for discussion
Technological support	•IT support for the ADAPT portal	

^a^Table reproduced with permission (Shepherd et al. 2019, The elusive search for success: defining and measuring implementation outcomes in a real-world hospital trial, *Frontiers in Public Health*) [26]

Services randomised to the *Enhanced strategy* received more prolonged, active engagement with the ADAPT team over the 12-month implementation period, including: monthly face-to-face meetings with the Lead Team, approximately 1 h in length, to discuss progress, identify training and support needs of local champions, highlight issues for attention and promotion to the wider team, and consider sustainability issues; additional awareness campaigns; and newsletters with progress updates sent to all staff with tailored strategies to address identified service-specific barriers and facilitators (an active approach).

### The ADAPT CP

After study launch, all sites implemented the ADAPT CP [17]. As per the ADAPT CP, participating patients completed anxiety and depression screening measures online and were allocated a severity step. Staff were contacted if severity was moderate or above and provided with recommended management options. Staff met with patients to confirm severity and triage them to appropriate management, checked referral uptake, reviewed progress and implemented re-screening.

### Outcome measures

Outcomes for this study have previously been described in detail [26].

#### Primary outcome: adherence

Adherence data for each cancer service was captured from the ADAPT Portal, supplemented by service medical record review. To address the ADAPT CP stepped care approach, we specified adherence as the percentage of all CP components (such as screening, triage, referral and re-screening) *appropriate to the patient’s level of anxiety/depression* (i.e. step allocation), undertaken at each screening episode, providing an adherence score of 0–100 (see Table 2). To address site tailoring of the ADAPT CP, delivery of CP components *by any appropriate staff* (individually defined at each site), was considered acceptable. Adherence to individual ADAPT CP components was also calculated.

**Table 2:** Adherence components for each anxiety/depression severity step

Anxiety/depression Step^a^ allocation	Screening	Triage	Referral made	Referral type approp	1st Follow-up	2nd Follow-up	Review psychological care status	Provider discharge treatment form	Re-screening	Total Components
1	✓	─	─	─	─	─	─	─	✓	2
2	✓	✓	✓	✓	✓	─	✓	✓	✓	8
3	✓	✓	✓	✓	✓	✓	✓	✓	✓	9
4	✓	✓	✓	✓	✓	✓	✓	✓	✓	9
5	✓	✓	✓	✓	✓	✓	✓	✓	✓	9

^a^*Step 1* = minimal anxiety/depression, *Step 2* = mild anxiety/depression, *Step 3* = moderate anxiety/depression, *Step 4* = severe anxiety/depression, Step 5 = very severe anxiety/depression

To provide a more clinically relevant measure of adherence (our primary outcome), we further defined a categorical outcome for each screening episode: (adherent ≥ 70% of key ADAPT CP components achieved; or non-adherent: < 70% of key ADAPT CP components achieved). The medical adherence literature cites optimal cut-offs of 80–90% [29, 30], however this cut-off would have resulted in substantial unbalance in sample sizes between the adherence outcomes, and limit statistical power to detect a difference in implementation arm. This is because managing anxiety and depression is complex and not the core business of oncology services, and so adherence can be low. Considering this, we chose a cut-off of 70% for this study.

#### Anxiety and depression step

The severity of anxiety and depression reported at each ADAPT CP screen was determined on the basis of screening scores. Participants completed the Edmonton Symptom Assessment System (ESAS-r) [31] or the Distress Thermometer (DT) [32] (each service chose one of these to use). Patients who scored ≥ 3 on the anxiety item or ≥ 2 on the depression item of ESAS-r or ≥ 4 on the DT were prompted to complete the Hospital Anxiety and Depression Scale (HADS) [33]. They were then allocated to an anxiety/depression severity step using published and consensus-derived cut-offs on the HADS (HADS score 0–3: step 1, HADS score 4–7: step 2, HADS score 8–10: step 3, HADS score 11–14: step 4 and HADS score ≥ 15: step 5) for minimal, mild, moderate, severe and very severe anxiety/depression respectively. Staff were alerted if patients scored at step 2 or above. Staff could adjust the step after a triage conversation with patients to determine the source and severity of their distress.

### Analysis

A multi-level mixed-effect logistic regression analysis was conducted using a binary outcome measure (adherent vs non-adherent) for the primary analysis and a multi-level mixed-effect multiple regression analysis for the continuous adherence outcome (0–100%) as the secondary analysis. In each analysis, implementation arm (Core vs. Enhanced) was fitted as a fixed-effect predictor. We included a fixed effect for step allocation (due to different number of components according to step allocation), and a random effect for the intercept, grouped hierarchically by persons (for repeated measures), and then site (due to the cluster randomisation). In an exploratory analysis, we tested for different effect sizes according to step allocation (by adding an interaction term between step allocation and implementation arm to the model).

## Results

Block randomisation resulted in 4 sites allocated to the Core arm, and 8 to the Enhanced (see Table 3 for site characteristics). Characteristics were evenly distributed across arms except for funding type, with all Enhanced arms being publically funded, while half the Core sites had a private or private/mixed funding model.

**Table 3:** Characteristics of participating sites (*n* = 12)

	Core (*n* = 4)	Enhanced (*n* = 8)
**Location**		
Major city	3	6
Inner regional	1	2
**Funding type**		
Public	2	8
Private or mixed	2	─
**Number patients seen per 3-month period**		
< 100	1	3
≥ 100	3	5
**Number departments included**		
1	3	2
2 or more	1	6
**Number tumour streams included**		
1 or 2	1	3
≥ 3	3	5
**FTE of psychosocial staff**		
0–4.9	3	6
≥ 5	1	2
**History of psychosocial screening in past 12 months**		
Yes	2	3
No	2	5

**Fig. 1:**
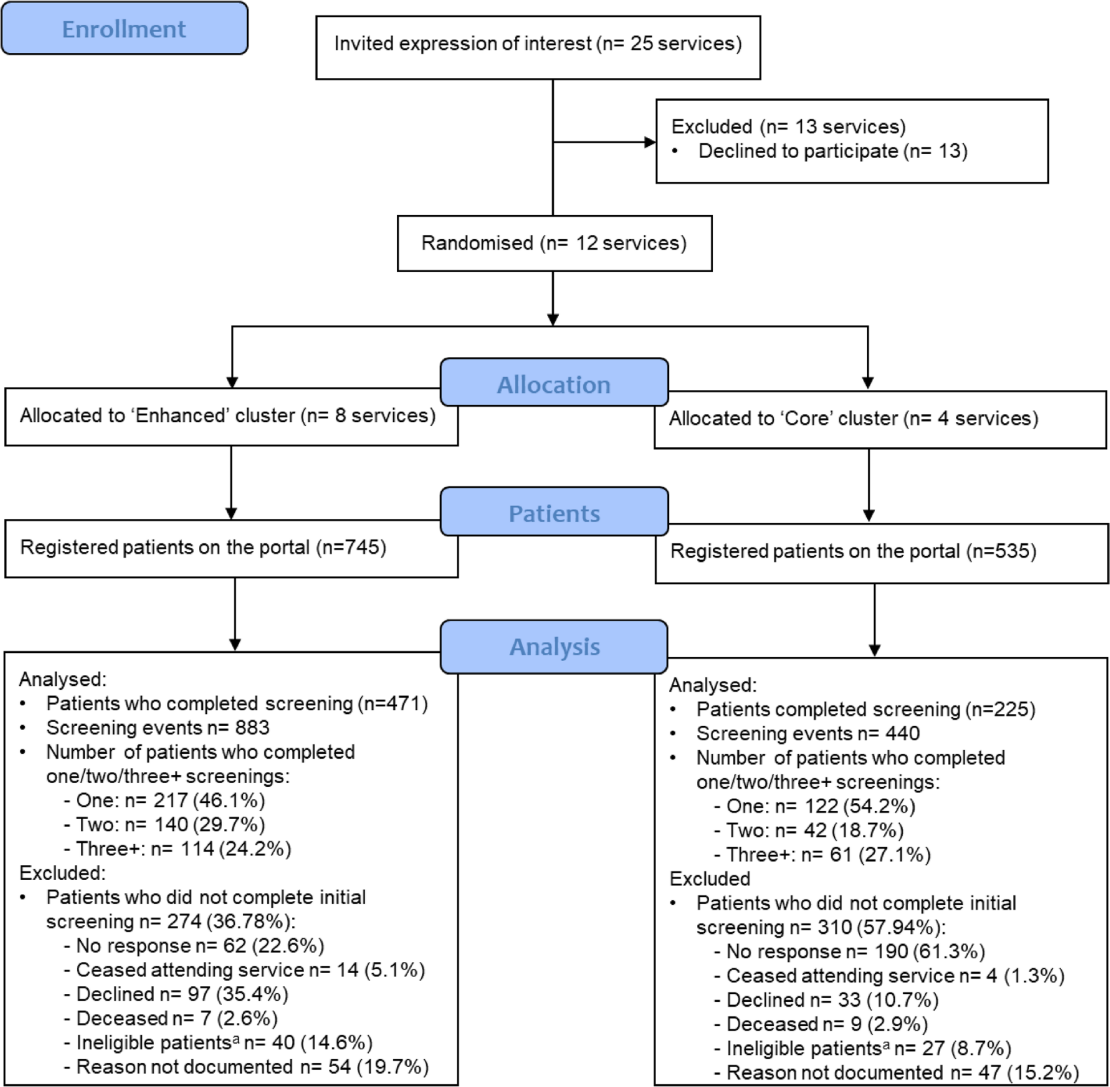
CONSORT flow diagram of progress for service recruitment, participant registrations, and screenings. ^a^ Patients ineligible if they i are unable to provide informed consent, ii have a cognitive impairment, iii have insufficient English to be able to complete the screening questions and do not have the aid of an interpreter or family member, iv did not receive a cancer diagnosis, or v did not screen within the 12-month study period

There were 1280 patients registered on the ADAPT Portal, 745 in the Core and 535 in the Enhanced services. Of registered patients, 696 (54%) went on to complete at least one screening, 63% in Core and 42% in Enhanced services. The most common reasons across arms for patients not screening were non-response to the screening invitation (41% of non-screeners), or patient decline (22% of non-screeners); specific reasons for non-response and decline were not recorded. As patients could complete more than one screen, a total of 1323 screening events (883 in Core and 440 in Enhanced services) were recorded (see Fig. 1).

There were slightly more females (62.6%) in the Core arm compared to the Enhanced arm (52.4%) (Table 4). Mean age of patients (63.4 years) was similar across arms. The most common cancer diagnoses were breast and genitourinary cancer. At the time of registration into the ADAPT CP, most patients had a diagnosis of stage 2/3 (regional spread) or stage 4 (distant spread) cancer. A higher (non-significant) proportion of patients completed two or more screenings in the Core (53.9%) than Enhanced (45.8%) arm.

**Table 4:** Demographic and clinical characteristics of patients who completed screening and the screening events

		Core	Enhanced	All total
Patients	**Gender**			
	Male	176 (37.4)	107 (47.6)	283 (40.7)
	Female	295 (62.6)	118 (52.4)	413 (59.3)
	**Age (in years)**			
	Mean (SD)	63.8 (12.6)	62.4 (12.9)	63.4 (12.7)
	**Cancer diagnosis**			
	Breast	149 (31.6)	45 (20.0)	194 (27.9)
	Gastrointestinal	123 (26.1)	73 (32.4)	196 (28.2)
	Genitourinary	55 (11.7)	10 (4.4)	65 (9.3)
	Gynaecological	41 (8.7)	10 (4.4)	51 (7.3)
	Haematological	9 (1.9)	27 (12.0)	36 (5.2)
	Head and neck	12 (2.6)	32 (14.2)	44 (6.3)
	Lung	51 (10.8)	22 (9.8)	73 (10.5)
	Melanoma and skin	14 (3.0)	4 (1.8)	18 (2.6)
	Neurological	1 (0.2)	0 (0)	1 (0.1)
	Sarcoma	7 (1.5)	1 (0.4)	8 (1.2)
	Cancer of unknown primary	4 (0.9)	0 (0)	4 (0.6)
	Other	5 (1.1)	1 (0.4)	6 (0.9)
	**Cancer stage**			
	Stage 0 in situ	4 (1.0)	3 (1.6)	7 (1.2)
	Stage 1 localised	49 (12.6)	38 (20.3)	87 (15.1)
	Stage 2/3 regional spread	177 (45.6)	63 (33.7)	240 (41.7)
	Stage 4 distant spread	158 (40.7)	83 (44.4)	241 (41.9)
	**Length of diagnosis (in days)**			
	Mean length of time from diagnosis to registration in the portal (mean, SD)	366.9 (699.6)	365.1 (1049.6)	366.3 (822.2)
	Mean length of time from diagnosis to first screening event (SD)	412.6 (702.2)	418.1 (1047.7)	414.3 (823.0)
	Missing date of diagnosis	6	19	25
	**Indigenous status**			
	Aboriginal but not Torres Strait Islander	25 (5.3)	6 (2.7)	31 (4.5)
	Torres Strait Islander but not Aboriginal	1 (0.2)	─	1 (0.1)
	Both Aboriginal and Torres Strait Islander	─	─	─
	Neither Aboriginal nor Torres Strait Islander	438 (93.0)	194 (86.2)	632 (90.8)
	Not stated/unknown	7 (1.5)	25 (11.1)	32 (4.6)
	**Number of screens completed**			
	1	217 (46.1)	122 (54.2)	339 (48.7)
	≥ 2	254 (53.9)	103 (45.8)	357 (51.3)
Screening events	**Initial screens**			
	Pre-triage anxiety/depression step^a^			
	Step 1	280 (59.5)	138 (61.3)	418 (60.1)
	Step 2	65 (13.8)	29 (12.9)	94 (13.5)
	Step 3	64 (13.6)	31 (13.8)	95 (13.7)
	Step 4	62 (13.2)	27 (12.0)	89 (12.8)
	Post-triage step			
	Step 1	306 (65.0)	149 (66.2)	455 (65.4)
	Step 2	54 (11.5)	30 (13.3)	84 (12.1)
	Step 3	38 (8.1)	25 (11.1)	63 (9.1)
	Step 4	15 (3.2)	11 (4.9)	26 (3.7)
	Post-triage step unconfirmed	58 (12.3)	10 (4.4)	68 (9.8)
	Step allocation following triage ^b^			
	Maintained	70 (52.6)	43 (55.8)	113 (53.8)
	Downgraded	63 (47.4)	32 (41.6)	95 (45.2)
	Upgraded	─	2 (2.6)	2 (1.0)
	**Subsequent screens**			
	Pre-triage step			
	Step 1	289 (70.2)	163 (75.8)	452 (72.1)
	Step 2	47 (11.4)	24 (11.2)	71 (11.3)
	Step 3	41 (10.0)	15 (7.0)	56 (8.9)
	Step 4	35 (8.5)	13 (6.1)	48 (7.7)
	Post-triage step			
	Step 1	297 (72.1)	171 (79.5)	468 (74.6)
	Step 2	31 (7.5)	18 (8.4)	49 (7.8)
	Step 3	26 (6.3)	6 (2.8)	32 (5.1)
	Step 4	10 (2.4)	3 (1.40)	13 (2.1)
	Post-triage step unconfirmed	48 (11.7)	17 (7.9)	65 (10.4)
	Step allocation following triage ^b^			
	Maintained	49 (65.3)	16 (45.7)	65 (59.1)
	Downgraded	26 (34.7)	18 (51.4)	44 (40.0)
	Upgraded	─	1 (2.9)	1 (0.9)

^a^*Step 1* = minimal anxiety/depression, Step 2 = mild anxiety/depression, *Step 3* = moderate anxiety/depression, Step 4 = severe anxiety/depression, *Step 5* = very severe anxiety/depression but no patients scored in this Step

^b^Excludes screening events where the pre-triage step allocation was not confirmed and those who screened as step 1 in the ADAPT Portal

Seventy-four percent of patients were categorised as having minimal or mild anxiety/depression after their first screen, with the remainder evenly distributed between steps 3–4 (there were no step 5 s). Following triage conversations, most step allocations were maintained or downgraded to a lesser step by staff in both Core and Enhanced arms, resulting in 63 at step 3 (9.1%) and 26 at step 4 (3.7%).

### Adherence

**Fig. 2:**
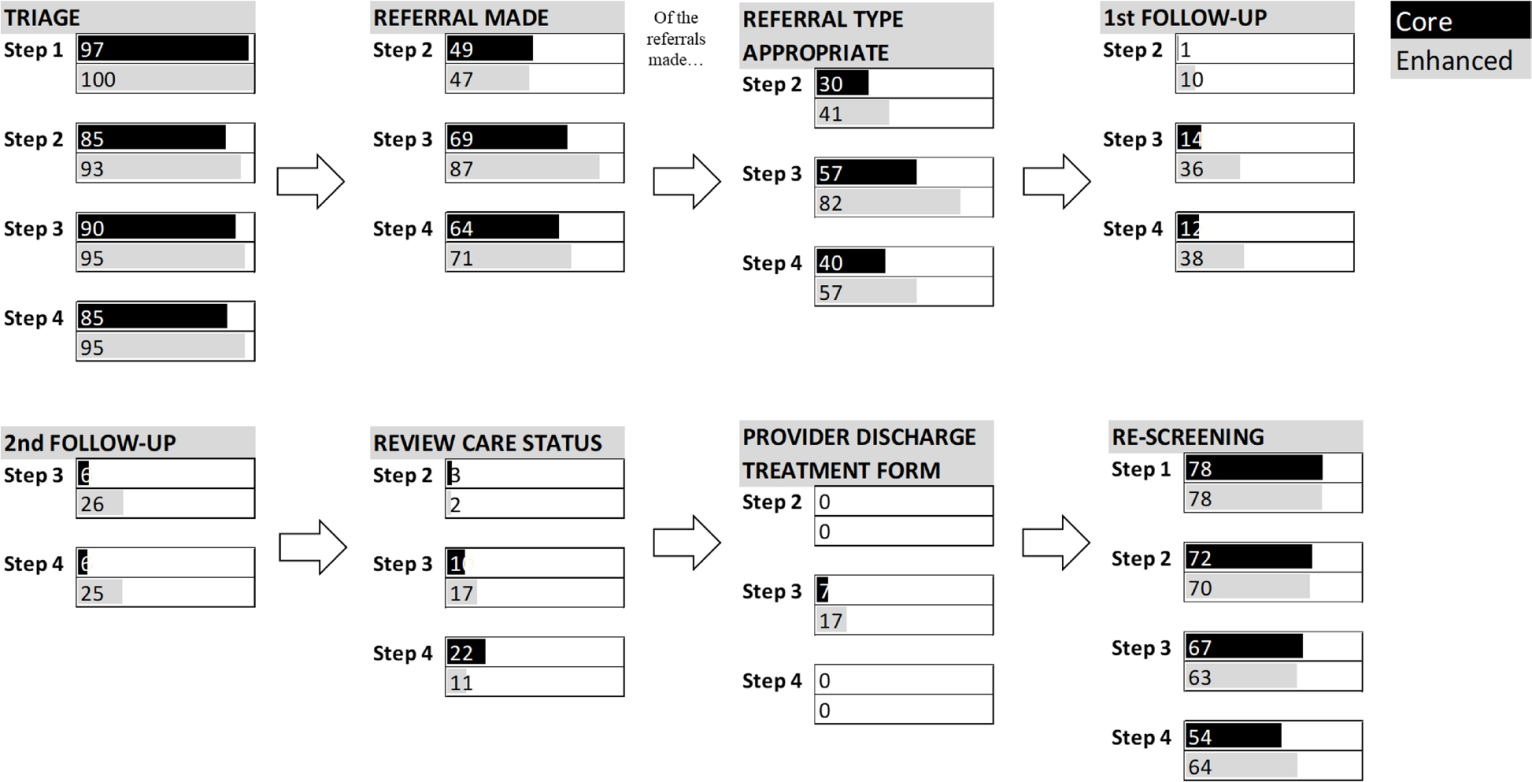
Adherence to individual components of the ADAPT pathway, in Core versus Enhanced arms, and by anxiety/depression Step^a^. ^a^Step 1 = minimal anxiety/depression, Step 2 = mild anxiety/depression, Step 3 = moderate anxiety/depression, Step 4 = severe anxiety/depression, Step 5 = very severe anxiety/depression but no patients scored in this Step

Adherence (as described above) was measured by scoring completion of individual ADAPT CP components, e.g. screening, triage, referral and re-screening. As data could be analysed only for participants who completed at least one screen, adherence for the initial screening component was by definition 100%. Adherence was high for triage (Core = 88%, Enhanced = 95%) and referrals being made (Core = 59%, Enhanced = 64%), and rescreening (Core = 75%, Enhanced = 75%), but poorer for ensuring referrals were appropriate (Core = 41%, Enhanced = 58%) and very poor for checking uptake (Core = 8%, Enhanced = 24%) and outcome of referral (Core = 6%, Enhanced = 25%) (see Table 5 and Fig. 2).

**Table 5:** Adherence for each component within the ADAPT CP presented by step allocation^a^ and implementation arm

	Step 1		Step 2		Step 3		Step 4		All total	
	Core	Enhanced	Core	Enhanced	Core	Enhanced	Core	Enhanced	Core	Enhanced
**Screening**										
*Adherent*										
Frequency	603	320	129	59	98	40	53	21	**883**	**440**
Percent	100.0	100.0	100.0	100.0	100.0	100.0	100.0	100.0	**100.0**	**100.0**
*Non-adherent*^b^										
Frequency									**0**	**0**
Percent									**0.0**	**0.0**
**Triage**										
*Adherent*										
Frequency	33	19	109	54	88	37	45	20	**275**	**130**
Percent	97.1	100.0	84.5	93.1	89.8	94.9	84.9	95.2	**87.6**	**94.9**
*Non-adherent*										
Frequency	1	0	20	4	10	2	8	1	**39**	**7**
Percent	2.9	0.0	15.5	6.9	10.2	5.1	15.1	4.8	**12.4**	**5.1**
**Referral made**^**c**^										
*Adherent*										
Frequency			63	27	68	34	34	15	**165**	**76**
Percent			51.2	53.5	30.6	12.8	35.9	28.6	**58.9**	**64.4**
*Non-adherent*										
Frequency			66	31	30	5	19	6	**115**	**42**
Percent			51.2	53.5	30.6	12.8	35.9	28.6	**41.1**	**35.6**
**Referral type**										
*Adherent*										
Frequency			39	24	56	32	21	12	**116**	**68**
Percent			30.2	41.4	57.1	82.1	39.6	57.1	**41.4**	**57.6**
*Non-adherent*										
Frequency			90	34	42	7	32	9	**164**	**50**
Percent			69.8	58.6	42.9	18.0	60.4	42.9	**58.6**	**42.4**
**Uptake 1**										
*Adherent*										
Frequency			1	6	13	14	6	8	**20**	**28**
Percent			0.8	10.3	13.8	35.9	12.2	38.1	**7.5**	**23.7**
*Non-adherent*										
Frequency			124	52	81	25	43	13	**248**	**90**
Percent			99.2	89.7	86.2	64.1	87.8	61.9	**92.5**	**76.3**
**Uptake 2**										
*Adherent*										
Frequency					6	10	3	5	**9**	**15**
Percent					6.3	25.6	5.7	25.0	**6.1**	**25.4**
*Non-adherent*										
Frequency					89	29	50	15	**139**	**44**
Percent					93.7	74.4	94.3	75.0	**93.9**	**74.6**
**Progress review**										
*Adherent*										
Frequency			3	1	7	5	8	1	**18**	**7**
Percent			2.6	2.0	10.1	17.2	21.6	11.1	**8.1**	**8.0**
*Non-adherent*										
Frequency			114	48	62	24	29	8	**205**	**80**
Percent			97.4	98.0	89.9	82.8	78.4	88.9	**91.9**	**92.0**
**Treatment contact**										
*Adherent*										
Frequency			0	0	2	5	0	0	**2**	**5**
Percent			0.0	0.0	6.9	17.2	0.0	0.0	**3.9**	**10.6**
*Non-adherent*										
Frequency			8	7	27	24	14	11	**49**	**42**
Percent			100.0	100.0	93.1	82.8	100.0	100.0	**96.1**	**89.4**
**Rescreening**										
*Adherent*										
Frequency	333	186	68	31	48	19	19	7	**468**	**243**
Percent	78.0	77.8	72.3	70.5	66.7	63.3	54.3	63.6	**74.5**	**75.0**
*Non-adherent*										
Frequency	94	53	26	13	24	11	16	4	**160**	**81**
Percent	22.0	22.2	27.7	29.6	33.3	36.7	45.7	36.4	**25.5**	**25.0**

^a^*Step 1* = minimal anxiety/depression, *Step 2* = mild anxiety/depression, *Step 3* = moderate anxiety/depression, *Step 4* = severe anxiety/depression, *Step 5* = very severe anxiety/depression but no patients scored in this Step

^b^This tables displays frequencies and proportions for those who have been allocated to a particular step. There are no values in this section since those who did not screen cannot be allocated to a step

^c^Values are provided only for components appropriate to each step

### Differences in adherence between arms

**Fig. 3:**
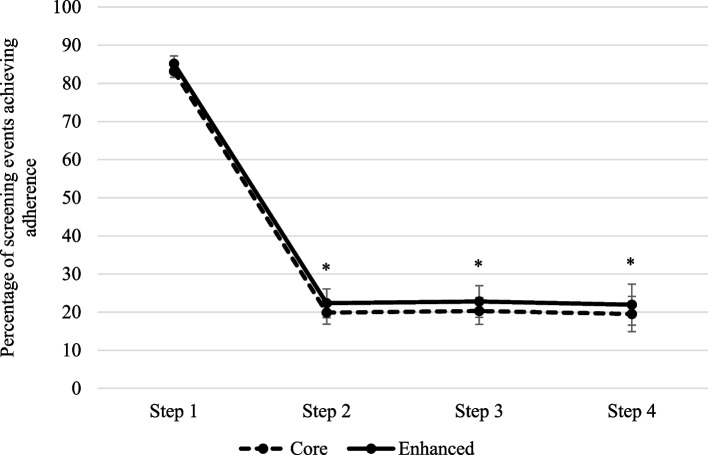
Adjusted mean differences in percentage of screening events for whom sites achieved adherence (binary outcome, > 70%) between arms. ^a^Step 1 = minimal anxiety/depression, Step 2 = mild anxiety/depression, Step 3 = moderate anxiety/depression, Step 4 = severe anxiety/depression, Step 5 = very severe anxiety/depression but no patients scored in this Step

There was no significant difference between arms in adherence (64% versus 66% for Core versus Enhanced arms, *p* = 0.35), when using the binary outcome variable of adherent versus non-adherent, controlling for persons (due to repeated screens) and site (due to cluster randomisation) (see Fig. 3 and Table 6). We conducted sensitivity analyses and results did not change from the non-significant finding using the 70% adherence cut-off. There was a significant difference in adherence according to anxiety/depression step (*p* < 0.001), with adherence lower for step 2 (OR = 0.05, 95% C.I. 0.03–0.09), step 3 (OR = 0.05, 95% C.I. 0.03–0.09) and Step 4 (OR = 0.05, 95% C.I. 0.02–0.10) where a greater staff response was required. The interaction term (implementation arm x step allocation) was non-significant (*p* = 0.11).

**Table 6:** Results with adherence as a binary outcome

Variable	Odds ratio (average difference to referent)	95% Confidence interval	*p* value	Adjusted means (%)^b^
**Implementation arm**			0.35	
Core	Referent			64.06
Enhanced	1.16	0.85–1.59		66.19
**Step allocation**^**a**^			< 0.001	
1	Referent			83.83
2	0.05	0.03–0.09		20.70
3	0.05	0.03–0.09		21.13
4	0.05	0.02–0.10		20.32

*Note*: As the interaction between implementation arm and step allocation was non-significant (*p* > 0.05), this table only presents the results of the main effects

^a^*Step 1* = minimal anxiety/depression, *Step 2* = mild anxiety/depression, *Step 3* = moderate anxiety/depression, *Step 4* = severe anxiety/depression, *Step 5* = very severe anxiety/depression but no patients scored in this Step

^b^ Adjusted mean differences in percentage of screening events achieving adherence (> 70%). Means have been adjusted for persons (due to repeated screens) and site (due to cluster randomisation)

**Fig. 4:**
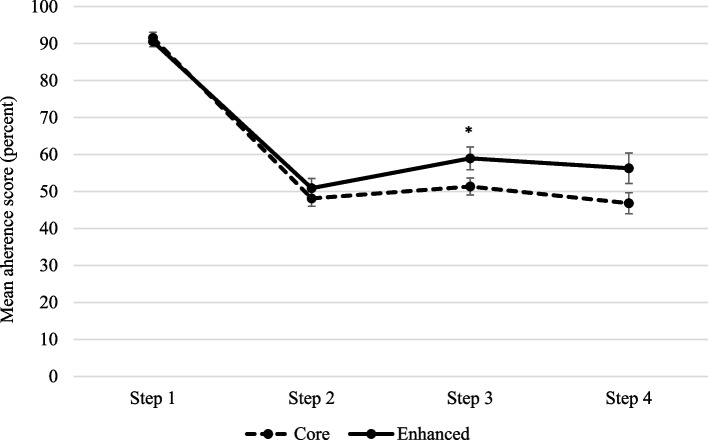
Adjusted mean differences in percent adherence (continuous outcome) between arms. ^a^Step 1 = minimal anxiety/depression, Step 2 = mild anxiety/depression, Step 3 = moderate anxiety/depression, Step 4 = severe anxiety/depression, Step 5 = very severe anxiety/depression but no patients scored in this Step

We repeated the analysis with adherence as a continuous variable (see Fig. 4 and Table 7). While similar results were found for main effects, here the interaction term (implementation arm x step allocation) was significant (*p* = 0.02), with adherence significantly higher for step 3 in the Enhanced arm (*p* = 0.048).

**Table 7:** Differences between arms in adherence (continuous outcome)

Variable	Mean adherence (95% CI)	Mean difference to referent^b^	95% Confidence interval	*p* value
**Step 1**^**a**^				
Core	91.5 (88.5–94.6)	Referent		
Enhanced	90.6 (87.7–93.4)	− 1.00	− 5.14–3.15	0.64
**Step 2**				
Core	48.1 (44.1–52.2)	Referent		
Enhanced	50.9 (45.8–56.0)	2.78	− 3.74–9.29	0.40
**Step 3**				
Core	51.4 (46.9–55.8)	Referent		
Enhanced	59.0 (52.9–65.0)	7.61	0.084–15.14	0.048
**Step 4**				
Core	46.8 (41.2–52.4)	Referent		
Enhanced	56.3 (48.2–64.4)	9.46	− 0.38–19.29	0.06
**Interaction between step allocation and implementation arm**				0.025

^a^*Step 1* = minimal anxiety/depression, *Step 2* = mild anxiety/depression, *Step 3* = moderate anxiety/depression, *Step 4* = severe anxiety/depression, *Step 5* = very severe anxiety/depression but no patients scored in this Step

^b^Means have been adjusted for persons (due to repeated screens) and site (due to cluster randomisation)

### Differences in individual adherence components between arms

There were significant differences between study arms for some adherence components, with higher adherence for the Enhanced arm when compared to the Core arm for triage (OR = 2.0, 95% C.I. 1.11–7.32), referral type (OR = 2.3, 95% C.I. 1.32–3.94), and check referral uptake (OR = 4.5, 95% C.I. 2.35–8.73). For all components, there was no significant interaction between implementation arm and step allocation (*p* > 0.05) in terms of adherence.

Across both study arms there were significantly more referrals made for step 3 and step 4 than step 2 (*p* < 0.01), and more appropriate referrals made for step 3 compared to step 2 (*p* < 0.001). Conducting a progress review after referral and rescreening were more likely to occur for step 3 and 4 than step 2 (*p* < 0.01).

## Discussion

Our primary objective was to determine if an Enhanced, ongoing ‘active’ implementation strategy throughout the first year of implementing a clinical pathway for anxiety and depression would lead to better staff adherence to the ADAPT CP than a Core, more ‘passive’ limited implementation strategy. We found no differences between the two implementation arms in adherence when we used either a binary outcome (≥ 70% of components adhered to, versus < 70%) or a continuous outcome. Our secondary objective was to examine the effect of step allocation (overall, and by implementation support arm) on total and component adherence scores. We found that step allocation was significantly associated with adherence; adherence was lower for higher (more severe) steps of anxiety/depression. When adherence was analysed as a continuous outcome only, there was also weak evidence for a difference (*p* = 0.048) between step allocation and implementation support arm. Adherence was significantly higher for step 3 (moderate) anxiety/depression in the Enhanced versus Core arm.

Our finding that implementation strategy as a main effect did not impact adherence is in contrast to the findings of Almatar et al. [34], who found that additional monthly feedback did improve adherence to a clinical pathway for community-acquired pneumonia over education alone. Notably, champions at Core arm sites did also receive (uncurated) monthly feedback, which may have reduced our ability to detect differences between the arms.

However, our cut-off for adherence, while based on similar studies, is somewhat arbitrary. When we used a continuous outcome, providing maximum power, and included the interaction term between implementation strategy and severity step, we found a marginally significant difference between arms for adherence to the ADAPT CP for patients with higher or more severe levels of anxiety and/or depression. Controlling for other potential confounders, staff in the Enhanced arm adhered to about 7.5 percentage points more components of the CP than those in the Core arm, for patients with step 3 anxiety/depression. While statistically non-significant due to considerable variability, there was a 10 percentage-point difference in adherence between arms for patients with step 4 anxiety/depression. Thus, the Enhanced implementation effort did appear to assist services to respond to patients with more severe anxiety/depression, who arguably need help more specialist care than those with minimal to mild anxiety/depression. However, this result must be interpreted with caution, given the marginally significant result.

These results provide some of the first empirical data on the extent of implementation effort required to ensure success in implementing health service change in the form of a novel CP. Our study suggests that even when great care has been taken to design and launch a new CP, adhering to principles described in implementation science frameworks, ongoing assistance during implementation facilitates greater staff adherence. While frameworks such as the Consolidated Framework for Implementing Research (CFIR) [16] do emphasise ongoing processes, particularly tracking progress and refining an intervention across time to simplify and enable execution, this longitudinal aspect of implementation has received relatively little attention in the implementation science literature to date [15, 16]. Cancer services in our Enhanced arm received ongoing progress reports; scheduled monthly meetings to discuss reports, identify and overcome barriers, identify training and support needs of local champions and deliver these, and highlight issues for attention and promotion to the wider team. In addition, ongoing awareness campaigns were run and regular newsletters with progress updates and tailored strategies to address identified service-specific barriers were sent to all staff. Further research is required to identify any additional strategies that could promote uptake of health service initiatives over time.

If longitudinal implementation effort is required to achieve health service change, adequate resourcing will be needed not only for initial effort, but for sustainment. Our own health economic data (paper under review) will inform appropriate planning. Unfortunately to date, it appears that health systems often fail to dedicate sufficient resourcing to integrate and sustain implementation efforts, particularly those focused on improving mental health [35]. Both funding and sufficient staffing are required during implementation of novel CPs. In Australia, as elsewhere, there are significant psycho-oncology workforce shortages, with few hospital positions allocated to this role.

All patients included in this data set had completed screening at least once. Subsequent adherence was highest across both arms of the study for triage (85–100% across anxiety/depression steps). Staff followed up concerning patient scores on screening measures, by having a discussion with them to clarify their concerns and needs. Adherence to re-screening, whilst lower than for triage, was also relatively high (54–78%). Screening for distress using patient-reported outcome measures has been promoted for many years [36, 37], so it is unsurprising that staff found these aspects of the ADAPT CP the easiest to adhere to, while triage is a clinical strategy with which most staff are familiar.

However, adherence to interim ADAPT CP steps (tracking whether patients took up referrals, were happy with care received and improved or required re-referral, and obtaining a treatment discharge summary) was poor. These components, laid out in the ADAPT CP, are particularly important for patients with more severe morbidity who require referral. Yet we observed a highly significant difference for the main effect of anxiety/depression step on adherence, with lower adherence overall for those with greater anxiety/depression.

While we did not directly assess reasons for this lower adherence to interim CP components in the current trial, we can speculate regarding causes. Oncology staff are likely less familiar with these components, than with initial screening. There may also be a lack of referral pathways, given psychosocial workforce limitations, and staff may therefore be referring out to community services with whom they have less established communication channels. Furthermore, these steps can require more time and be frustrating if the specialist staff to whom patients have been referred respond slowly or not at all to requests for information and discharge summaries. Integrating psychological care related to cancer diagnosis that occurs outside of the cancer centre could be complex and require careful planning and system changes. Staff may need targeted support in integrating these CP components within existing workflow processes.

It is also possible that these steps occurred but were not well documented in either the ADAPT Portal or patient records. However, without documentation, steps to address non-uptake of referral or need for new referral may not consistently occur, impacting patient outcomes [5, 13]. An advocacy effort to promote these aspects of quality care is now needed, given the success of earlier advocacy efforts to entrench screening into routine care.

Our study had significant strengths. We employed a cluster randomised controlled trial design, that allowed us to clearly compare two implementation strategies. We recruited diverse oncology services across urban and regional areas, and public and private health care systems. We collected detailed data on staff adherence to each of the CP components and controlled for potential site and patient confounders. Reporting complied with CONSORT reporting standards [38] (see Additional file 1).

The study also had limitations. While our study compared a Core implementation strategy with an Enhanced one that provided implementation support across one year, it is not clear what intensity of implementation support is required to achieve improved adherence. Perhaps less ongoing support may have been sufficient, or more would have achieved an even better outcome. Future studies could compare more or less intense implementation efforts, compare length of supported needed and tease out which strategies were most helpful.

We were not able to obtain accurate data on the proportion of all eligible patients who were invited by staff to participate in the ADAPT CP. The implementation strategy delivered may have effected the level of staff commitment, time and energy devoted to encouraging patients to screen for anxiety and depression, and this should be explored in future trials.

An important consideration is how much statistical power we had to detect an effect if one was present. This information is best captured in the bounds of the confidence intervals for the effect sizes. Although there was no effect of implementation arm on adherence as a binary outcome (Table 6), the reasonable range of possible effect sizes that the implementation could have had is an odd’s ratio between 0.85 and 1.59. Thus it is possible that we failed to detect an effect (within the confidence interval bounds) that was present. However, as there are very few implementation studies in Psycho-Oncology on which to base effect size estimates, it is difficult to interpret this potential effect size with confidence. This demonstrates the need for future work in this area to provide more precise estimates of effect sizes.

Our focus was on services adherence to the ADAPT CP. Due to small numbers (*n* = 12), it was difficult to adjust for small differences between services across study arms, such as in funding type, and this may have resulted in some bias in the results.

The ADAPT Portal was not able to integrate with the electronic medical records (EMRs) used in participating hospitals, due to diversity of systems used across services. This likely reduced staff compliance in recording their actions within the Portal in line with instructions. Thus, adherence to the ADAPT CP may have been higher than we could detect. However, we did conduct exhaustive medical record reviews to supplement the portal data and ensure our adherence data was as accurate as possible. Nevertheless, future studies would benefit from integrating data collection methods within existing hospital EMR systems.

## Conclusions

In summary, this study suggested that an Enhanced implementation strategy that supported staff over 1 year may support staff to be more adherent to a clinical pathway for anxiety/depression, at least for patients with more severe anxiety/depression. Wensing and colleagues [39] noted that with the increasing pace of scientific discovery, and the number of new programs and technologies being introduced into the health care system each year, successful and efficient integration of these interventions into routine care is increasingly required. Our data suggest that commitment of sufficient staff and resources to support staff during implementation efforts will increase our success in incorporating evidence into patient care and improving outcomes.

## Supplementary Information

**Additional file 1.** CONSORT Reporting Standards Checklist.

## Data Availability

The datasets used and/or analysed during the current study are available from the corresponding author on reasonable request.

## Abbreviations

ADAPT: The Anxiety and Depression Pathway Program. A Translational Program Grant: A Sustainable and supported clinical pathway for managing anxiety and depression in cancer patients
ADAPT CP: Clinical Pathway for the Screening, Assessment and Management of Anxiety and Depression in Adult Cancer Patients: Australian Guidelines
CP: Clinical pathways
PARiHS: Promoting Action Research in Health Services framework
CFIR: Consolidated Framework for Implementing Research
ADAPT Portal: Web-based system to operationalise the Clinical Pathway for the Screening, Assessment and Management of Anxiety and Depression in Adult Cancer Patients: Australian Guidelines
CRCT: Cluster randomised controlled trial
NSW: New South Wales
ESAS-r: Edmonton Symptom Assessment System
DT: Distress thermometer
HADS: Hospital Anxiety and Depression Scale
EMRs: Electronic medical records
