# Supplementary material for: Effect of core versus enhanced implementation strategies on adherence to a clinical pathway for managing anxiety and depression in cancer patients in routine care: a cluster randomised controlled trial
Source: Implement Sci. 2023 May 22;18:18. doi: 10.1186/s13012-023-01269-0 (PMC10204284; doi:10.1186/s13012-023-01269-0)
Supplement: Supplementary file 1 — Additional file 1. CONSORT Reporting Standards Checklist. [file 13012_2023_1269_MOESM1_ESM.docx]

Additional File 1. CONSORT Reporting Standards Checklist

| Item | Item description | Page no. where this is covered. |
| --- | --- | --- |
| **Title** | Identification of study as cluster randomised | 1 |
| Trial design | Description of the trial design (e.g. parallel, cluster, non-inferiority) | 6 |
| **Methods** |  |  |
| Participants | Eligibility criteria for clusters | 6-7 |
| Interventions | Interventions intended for each group | 8-9 |
| Objective | Specific objective or hypothesis | 5-6 |
| Outcome | Whether the primary outcome pertains to the cluster level, the individual participant level or both | 9 |
| Randomisation | How clusters were allocated to interventions | 8 |
| Blinding (masking) | Whether or not participants, care givers, and those assessing the outcomes were blinded to group assignment | 8 |
| **Results** |  |  |
| Recruitment | Trial status | 11 |
| Numbers analysed | Number of clusters analysed in each group | 11 |
| Outcome | Results at the cluster or individual participant level as applicable for each primary outcome | 12-13 |
| Harms | Important adverse events or side effects | n/a |
| **Conclusions** | General interpretation of the results | 14-18 |
| **Trial Registration** |  | 3 |
| **Funding** | Source of funding | 24 |
